# Models of turfgrass seed germination related to water content

**DOI:** 10.1371/journal.pone.0204983

**Published:** 2018-10-08

**Authors:** Ying Jiang, Derong Su

**Affiliations:** 1 Guizhou Botanical Garden, Guiyang, Guizhou, China; 2 Grassland Resources and Ecology Research Center, Beijing Forestry University, Beijing, China; College of Agricultural Sciences, UNITED STATES

## Abstract

Turfgrasses have been widely utilized by humans to enhance the environment for more than several centuries. Seed germination is of great importance in the development of the turfgrass industry. In this study, the seed germination models of the responses of three types of turfgrasses to water were studied. The results indicated that irrigation water was mainly related to the variation of seed volume rather than a fixed value in this experiment. The maximum *k* value of tall fescue, Kentucky bluegrass and perennial ryegrass is 1.1548, 1.6946 and 4.0940, respectively. The optimal value of *k* is 1.0617 for tall fescue, 1.4610 for Kentucky bluegrass, and 1.6614 for perennial ryegrass. Perennial ryegrass seeds are more sensitive to water than those of tall fescue and Kentucky bluegrass, and this turfgrass is the fastest to reach the maximum value of the germination rate. Based on the results from the present experiment, the seed germination function can describe the response of turfgrass seed germination to external water content variation and their sensitivities. The function obtained could be used to perform quantitative studies on the dynamic changes of seed germination under different water conditions that will contribute to improved predictions of the optimal fitting curves of the germination over a range of water.

## Introduction

Turfgrasses are widely used throughout the world to protect our environment, and they have been an issue of major national and international importance to modern societies for many centuries [1]. Seed germination is an important step in the plant life cycle, and seeds can only germinate normally in an appropriate ecological environment [2]. Water is a prerequisite for seed germination since seeds must absorb a certain amount of water to germinate [3]. A seed germination bed, as a place to obtain water, definitively affects seed germination by controlling the water content; if there is too little water, the seeds will not germinate without reaching the minimum water requirement; however, when there is too much water, factors such as hypoxia will reduce the germination rate [4].

Within a certain range of water content, the seed germination rate is significantly positively correlated with water supply [5]. If the supply is inadequate, the seed germination will be restricted by the water content [6]. Previous studies suggested that seed germination can occur after the completion of imbibition and absorption, that is, the germinating seeds need time to prime, and this only occurs when the seeds absorbed water for a period of time [7,8]. The chemical composition of the seeds as well as the membrane permeability, seed size, 1000-grain weight, seed dormancy, temperature, oxygen, carbon dioxide, light, salinity, and water depth will affect the rate of water absorption and the water use efficiency of the seeds as well as their interactions with water during seed germination [9]. Research has indicated the minimum water requirement for seed germination in various plants using different modeling methods limited to different launch conditions. Research has also provided several models and empirical formulas, and the hydrothermal threshold model has been widely used to describe the temperature and water potential effect on seed germination [10]. In these models, researchers studied crop seeds based on the hydrothermal time (HTT) and virtual osmotic potential (VOP) models. These models assume that the variable conditions of the water potential depend on the current water environment. Therefore, when the difference value between the water and osmotic potentials exceeds a fixed threshold, the seed radicle will grow. Assessing the effect of the promotion of seed growth to establish the precise model would help to predict the variables of temperature and water potential on the influence of germination, which will advance the predicted simulation of seed priming time, which is conducive to seed germination, seedling emergence and seedling growth, and increase crop yields [11]. However, previous studies largely focused on prediction and improved the methods of seed priming time, ignoring the role of the external water environment during the changes of water content, such as excessive evapotranspiration due to a shortage of water or insufficient irrigation [11,12].

By simulating water stress conditions, it is possible to study the biochemical and physiological processes involved in the drought resistance of plants, which are similar to the effect of external water content variation. As a common herbaceous ground cover plant, turfgrass is a plant community that is often used in mixed sowing (different species or varieties). The relative germination time and germination capacity of each turfgrass plant population will play a key role in the dynamic competition of the community structure [13,14]. The extremely sensitive reaction of turfgrass to water suggests that their adaptability is closely related to the response of the water content variation during turfgrass seed germination and emergence. Considering the characteristics of water parallel diffusion in layered soil under subsurface drip irrigation, it can maintain relatively stable and uniform soil water contents in high-density planted turfgrass.

The objectives of this study were to study the seed germination rate under different water conditions, establish models between seed germination and water content, provide valuable information to evaluate the relationships between turfgrass seed germination and soil water content, and, finally, provide a theoretical basis for the application of subsurface drip irrigation to turfgrass.

## Materials and methods

### Theoretical considerations

Under suitable conditions, seeds absorb water and begin to germinate [15]. Seeds require external water for this process. At the same time, the external water may change due to environmental reasons. When the water is saturated, the seeds will quickly germinate. Subsequently the instantaneous germination rate will decrease as it reaches the optimal level, while the cumulative seed germination rate will gradually attain its maximum value. Considering that the seeds are not infinitely sprouting in the limited space and resources, when the germination rate reaches a certain point, the seeds will inhibit their growth. Therefore, it can be assumed that the environment can support the maximum of cumulative seed germination rate designated *a*, while *y* represents the current cumulative germination rate. Therefore, *a* − *y* is the germination rate left which the environment can support, and the changes in germination rate positively correlate with these two items. Thus:

$$\frac{dy}{dt}=r(a-y)y$$ (1)

where t is the germination time, and r is a positive constant. Separation of the variables and the integral for the Eq (1) results in:

$$y=\frac{a}{1+e^{-ra(t-c)}}$$ (2)

where *c* is a constant term arising from integration. The deformation to Eq (2) leads to:

$$y=\frac{a}{1+e^{-k(t-t_{c})}}$$ (3)

where *k* is a positive constant, and *k* expresses the sensitivity of the reaction of the seeds to environmental conditions. While the other conditions remain constant, *k* relates to the environment water, and *t*_*c*_ is the time when the instantaneous germination rate gains the maximum value. *k* = *ar*, Thus, Eq (1) becomes:

$$\frac{dy}{dt}=\frac{k}{a}(a-y)y$$ (4)

Seeking its second derivative:

$$\frac{d^{2}y}{dt^{2}}=\frac{k}{a}\frac{dy}{dt}(a-2y)$$ (5)

as $\frac{dy}{dt}\geq 0$, when $\frac{d^{2}y}{dt^{2}}=0$, then $\frac{dy}{dt}\neq 0$, $y=\frac{a}{2}$; this is the maximum point of the instantaneous germination rate.

**Fig 1:**
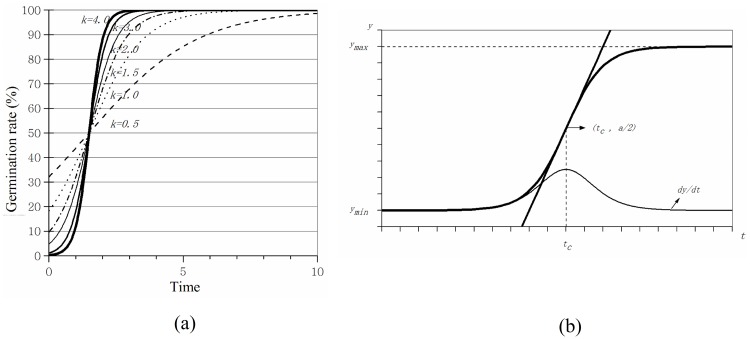
Seed germination function (A) and the characteristics of curves (B).

The changes in the seed germination function and the characteristics of the curves are shown in Fig 1. When the seeds begin to germinate, the instantaneous germination rate (slope of the curve) will increase, and the cumulative germination rate also increases (Fig 1A). The slope increases with *k*. The time it takes to achieve the maximum cumulative germination becomes shorter as the *k* increases. From Fig 1B, it can be concluded that when $y=\frac{a}{2}$, results in *t = t*_*c*_, the instantaneous germination rate will reach its maximum and start to decrease. The cumulative seed germination rate will gradually reach its maximum.

### Materials

The seeds of the three turfgrasses used in this study were obtained from the Beijing Clover Technical Development Center. The detailed information of these seeds is listed in Table 1.

**Table 1:** Turfgrass seeds used in the study.

Species	Variety	1000-Grain weight	Moisture
Tall fescue (*Festucaarundinacea*Schreb.)	Jaguar 3	2.330 g	9%
Perennial ryegrass (*Loliumperenne* L.)	High Cap 2	1.710 g	11%
Kentucky bluegrass (*Poapratensis* L.)	Midnight	0.320 g	8%

### Analytical procedures

#### Determination of the seed volumes

To test the average volume of the seed of each species, the seed specific gravity of each species was first determined. A total of 100 seeds of each species was randomly collected and weighed in three replicates and placed in a flask containing 50% ethyl alcohol. The flasks were shocked to exhaust the air (at 20 °C). The specific gravities of each species were calculated using the following formula:

$$S=\frac{W_{1}}{W_{2}+W_{1}-W_{3}}\times G$$ (6)

where *W*_1_ is the weight of 100 seeds of each species (g); *W*_2_ is the weight of flask with 50% ethyl alcohol (g); *W*_3_ is the weight of flask with ethyl alcohol and seeds (g), and *G* is the proportion of 50% ethyl alcohol. The volume of the seeds (*V*) was calculated using the following formula:

$$V=\frac{W}{S}$$ (7)

where *W* is the average weight of seed, and *S* is the specific gravities of the seed.

#### Determination of the seed equilibrium water content

One hundred seeds of each species were weighed and wrapped with gauze (three replicates). The seeds were placed in a thermostatic container at 25 °C with the appropriate amount of water to imbibe it and weighed after centrifugation at a 12 h-interval until their weight would not change. The seed equilibrium water content (*f*_*e*_)was calculated using the following equation:

$$f_{e}=\frac{EW-DW}{DW}$$ (8)

where *EW* is the equilibrium water weight (g), and *DW* is the dried weight of seeds.

#### Response of the seed germination to irrigation

One hundred seeds of each species were randomly chosen (three replicates). The volume of a 90 mm (diameter) Petri dish was first determined, and the weight of the seeds was calculated under the same volume. Finally, we weighed the dish which was covered with a layer of the seeds. The water content of a soaked layer of the seeds (*Y*_*s*_) was calculated as:

Ys=W5W4×V2 (9)

where *W*_4_ is the seed weight (g) of volume size of the Petri dish; *W*_5_ is the weight (g) of a layer seed covered Petri dish, and *V*_2_ is the volume of the Petri dish (mL).

The seeds were placed in the Petri dishes whose volume has been determined. Irrigation was conducted at ten levels (*y*_*s*_ × 10%, *y*_*s*_ × 20%, *y*_*s*_ × 30%, *y*_*s*_ × 40%, *y*_*s*_ × 50%, *y*_*s*_ × 60%, *y*_*s*_ × 70%, *y*_*s*_ × 80%, *y*_*s*_ × 90% and *y*_*s*_ × 100%). The Petri dishes were placed in a light and constant temperature incubator. The temperature was controlled at 25±1 °C, and the light was controlled at 12 h light / 12 h dark. These conditions were close to the optimal growth condition of the three turfgrasses studied. The radicle breaking through the seed coat was selected to be the germination standard, and the germination status was monitored and recorded at approximately 24-hour intervals. At the same time, each Petri dish was weighed to determine the evapotranspiration, and the loss of water caused by measurements was added. The observations continued until the germination rate remained the same for a week. The germination was assessed based on the seed germination rate and germination priming time, which was the time from sowing to the first seed germinated (days).

## Results

### Imbibition and equilibrium water of seed germination

**Fig 2:**
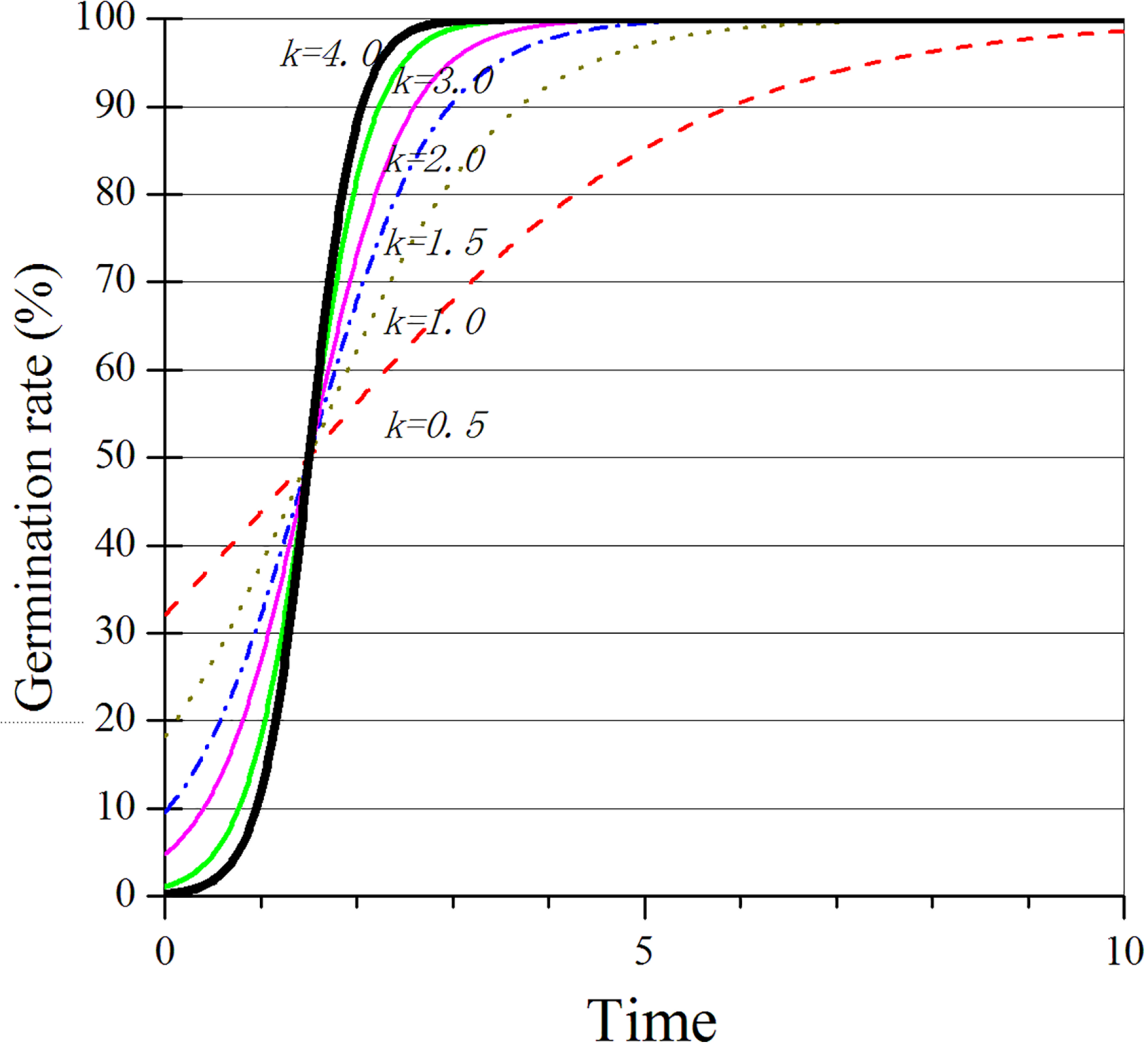
The change of external water of three turfgrasses.

The change in the external water minimum value (variation rate) of the three turfgrasses gradually decreased under the different irrigation standards (Fig 2). The external water did not change along with the increase in the irrigation water content. However, the variation rate decreased. This result helped to maintain the stability of the external environment water. It should be noted that the irrigation water in this experiment was primarily relative due to the variation of the seed volume rather than a fixed value. Thus, during practical applications, the seed germination is primarily determined by the water use efficiency rather than the water quantity. For example, if there is excessive evapotranspiration, the final storage capacity of the water will be low. The water content helped the seeds to germinate and improved the germination rate only when it remained stable to supply the water for a long time [16]. Therefore, when choosing different irrigation methods, it should be initially considered that the external water changes are smaller over a long period (irrigation cycle) and maintain high water use efficiency. As shown in Fig 1, there was no significant discrepancy among the three turfgrasses studied, and they share a similar variation tendency.

The water balance of the plant communities could be expressed by the water equilibrium equation [17]. There is only a small difference in the form of specific performance. In this experiment, irrigation is assumed to be the only water input for seed germination; thus, the water input (irrigation water) is equal to the loss of evapotranspiration water. However, when the quantity of the irrigation water is more than evapotranspiration or is too low to meet the growth needs of seeds, water storage would increase or decrease to maintain the water balance. Therefore, the irrigation water quantity equilibrium equation is:

$$W_{I}=W_{S}(t)+W_{E}(t)$$ (10)

where *W*_*I*_ is the irrigation water quantity, *W*_*S*_ is the variation of water storage, and *W*_*E*_ is the evapotranspiration water quantity.

Under stable external environmental conditions, such as when the temperature and humidity are relatively constant, the variation of evapotranspiration water is considered a constant, namely:

$$W_{E}\left(t\right)=vt$$ (11)

where *v* is a constant to indicate the evapotranspiration rate, and *t* is time.

Therefore, when the irrigation water remains stable, the seed germination mainly depends on the water storage capacity, and the *W*_*S*_ consists of the seeds imbibing water (*f*) and the external environment water (*φ*)which is not absorbed, namely:

$$W_{S}=f(t)+\varphi (t)$$ (12)

*f* can be expressed as a percentage of the dry weight of seeds. At the time of seed germination, there is an equilibrium between the seed imbibing water and the external environmental water, which is the maximum capacity of the seeds to imbibe water based on the seed itself and the environmental conditions, which is the seed equilibrium water content. Assuming that the water imbibition rate only depends on the current seed water content and equilibrium water content (*f*_*e*_), thus:

$$\frac{df}{dt}=L(f_{e}-f)$$ (13)

where *L* is the imbibition exponential coefficient of the seed; thus, we integrate Eq (13):

$$f=f_{e}(1-e^{-Lt})$$ (14)

When the seed imbibes water, the seed water content does not start from zero; however, from a fixed value *f*_*p*_ (initial water content), when *t* = 0, *f* = *f*_*p*_, as *t* infinity, *f* = *f*_*e*_, then the Eq (14) can be changed to:

$$f=f_{p}+(f_{e}-f_{p})(1-e^{-Lt})$$ (15)

where *L* is a constant related to the seed size and the external environment water. Since the same seeds are in each Petri dish, the impact of volume could be ignored, and the *L* is affected by the external environmental water. Then:

$$\varphi (t)=W_{I}-W_{E}(t)-f(t)$$ (16)

The seed germination occurred after the completion of imbibition and absorption. Therefore, when *t* = 0, *φ*(*t*) = *W*_*I*_, this is the maximum value of the water in the external environment. When *f* = *f*_*e*_, the minimum value appeared, *φ*(*t*) = *W*_*I*_ − *v*_*t*_ − *f*_*e*_. The same species in similar environments, *φ*(*t*) is mainly affected by the irrigation water *W*_*I*_, which indicates that the irrigation water content decides the rate of seed germination.

### Seed germination rate and equilibrium water

Population growth is restricted in a limited space and resources. When the population is excessive, it would restrain the growth. Thus, when the water varies, the germination rate also differs. In practice, Eq (3) often does not accurately determine the instantaneous time *t*_*c*_ when the germination rate reaches the maximum value, which is due to the germination rate increasing (Δy) maintaining a certain level of growth over a certain time interval. Therefore, the Δy of the two observation points may be similar or equal to the condition. If using the time (*t*_*m*_) to observe, and the maximum value of Δy simply corresponds to *t*_*c*_, it would lead to a large error for *k*. However, in the observations, it was determined that *t*_*c*_ must be near *t*_*m*_, and as shown in Fig 1B, the germination curve is approximately linear in the vicinity. This result would indicate that one of the two observation points, which are before or after *t*_*m*_ and gains a larger Δy, takes *t*_*c*_ as the average value of the larger Δy point and *t*_*m*_, thus simplifying the model:

$$t_{c}=\{\begin{array}{cc} (t_{m}+t_{m-1})/2, & \Delta y_{m-1}>\Delta y_{m+1}\\ t_{m}, & \Delta y_{m-1}=\Delta y_{m+1}\\ (t_{m}+t_{m+1})/2, & \Delta y_{m-1}<\Delta y_{m+1} \end{array}$$

This test, with Δ*t* = 1(time recorded every 24 h), results in:

$$t_{c}=\{\begin{array}{cc} t_{m}-0.5, & \Delta y_{m-1}>\Delta y_{m+1}\\ t_{m}, & \Delta y_{m-1}=\Delta y_{m+1}\\ t_{m}+0.5, & \Delta y_{m-1}<\Delta y_{m+1} \end{array}$$

where $\frac{dy}{dt}$ is the slope; when the increment Δ*t* is close to 0, then the slope is approximately equal to $\frac{\Delta y}{\Delta t}$; with equal interval sampling, *t*_*c*_ can be evaluated based on Δ*y*; in the model simplification, because the increasing value of the two sampling points were similar, it can determine that the maximum value is between the two points and the practice of *t*_*c*_, whose accuracy has been able to meet the demand of this test.

**Fig 3:**
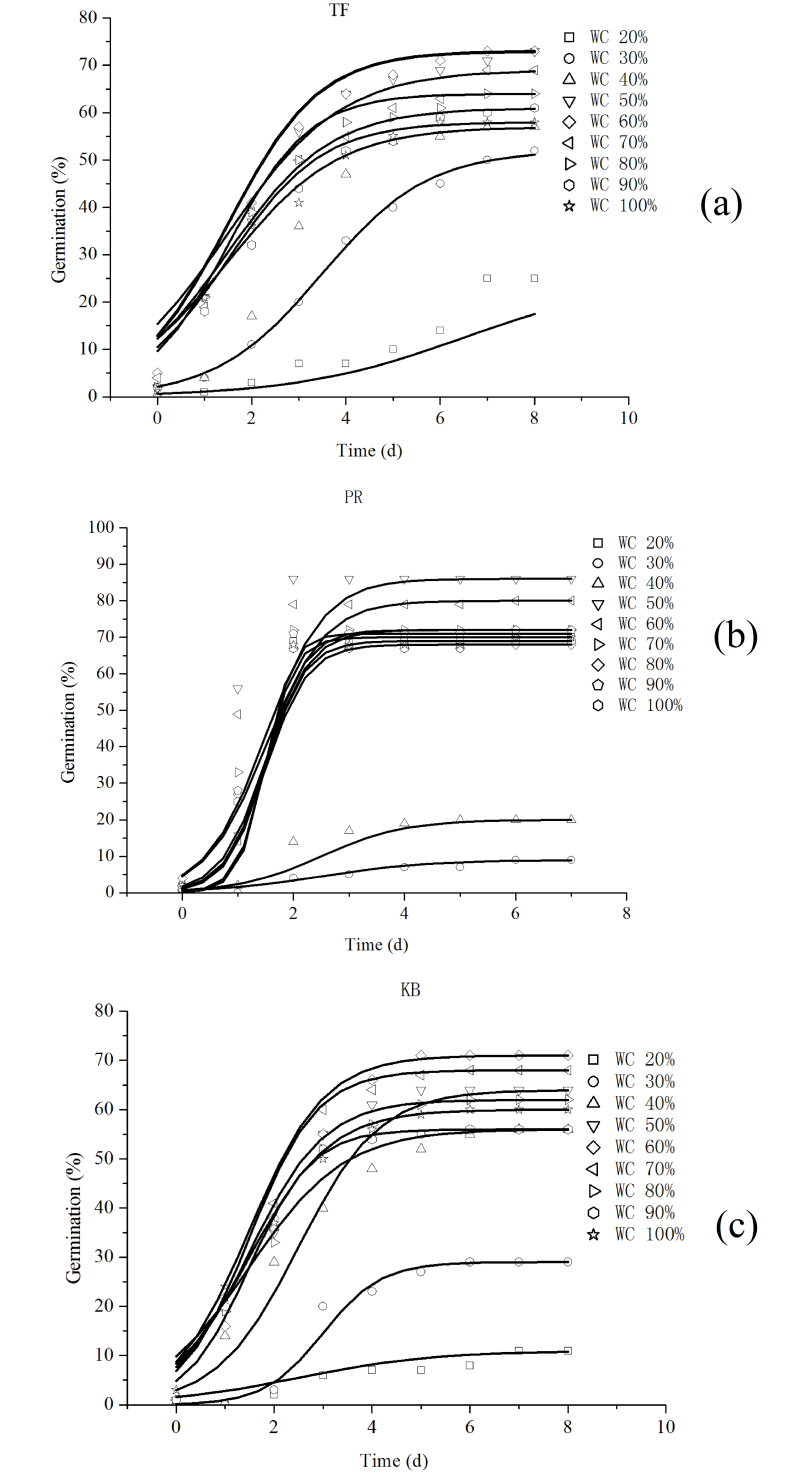
Mathematical model of the germination rate for three turfgrasses under study. (A) Tall fescue (TF), (B) Perennial ryegrass (PR) and (C) Kentucky bluegrass (KB).

The variations of the cumulative germination rate of the three turfgrasses under different irrigation levels are shown in Fig 3. The cumulative germination rate of all the three turfgrasses shows a “slow-fast-slow” change rule along with the water level, and the germination rate of different kinds of turfgrass seeds with varying water content all meet the same function. At the same time, when the amount of water increases to a certain degree, the cumulative germination rate gradually tends to slow and exhibits a downward trend. Thus, the existence of the optimal amount of water can make the seed reach the maximum germination rate; however, when it exceeds the boundary, it will inhibit the germination of the seeds.

### Factor of water sensitivity

The parameters and original data for the simulation in the seed germination function are listed in Table 2.

**Table 2:** Model parameters of different turfgrasses.

Species	Model parameters	Water content (%)									
		10	20	30	40	50	60	70	80	90	100
Tall fescue	a (%)	0.00	25.00	52.00	57.00	73.00	73.00	69.00	64.00	61.00	58.00
	t_c_(d)	0.00	6.50	3.50	1.50	1.50	1.50	1.50	1.50	1.50	1.50
	k	0.00	0.56	0.90	0.86	1.03	1.03	0.83	1.15	0.92	1.00
	R^2	0.00	0.71	1.00	0.80	0.94	0.97	0.93	0.97	0.94	0.91
Perennial ryegrass	a (%)	9.00	20.00	86.00	80.00	72.00	68.00	72.00	69.00	70.00	71.00
	t_c_(d)	2.50	2.50	1.50	1.50	1.50	1.50	1.50	1.50	1.50	1.50
	k	1.04	1.34	1.88	1.87	2.47	2.62	2.76	2.80	3.79	4.09
	R^2	0.94	0.86	0.75	0.78	0.89	0.92	0.93	0.94	0.98	0.99
Kentucky bluegrass	a (%)	0.00	11.00	29.00	56.00	64.00	71.00	68.00	62.00	56.00	60.00
	t_c_(d)	0.00	2.50	3.00	1.50	2.50	1.50	1.50	1.50	1.50	1.50
	k	0.00	0.72	1.69	1.03	1.21	1.31	1.45	1.30	1.56	1.21
	R^2	0.00	0.80	0.97	0.93	0.89	0.95	0.99	0.97	0.99	0.99

The results indicated that *k* reflected the difference of the sensitivities of the different seed species to external water content. If *k* increases, the cumulative germination rate changes more quickly with time. Thus, the instantaneous germination rate (the slope of the curve) changes more. Considering the relationship between *k* and the water, there is an assumption that when the seed imbibition and absorption of water are completed but the seeds have not started to germinate, *k* = 0; before the change rate of *φ*(*t*) reaches its optimal value, *k* increases as the irrigation water content increases; and when the change rate of *φ*(*t*) fluctuates around the optimal value (decrease or increase), *k* will decrease. The regression equations based on the data measured or the three turfgrasses are (Tall fescue, Eq 17; Perennial ryegrass, Eq 18; and Kentucky bluegrass, Eq 19):

$$k=-0.0242\varphi^{2}+0.3437\varphi -0.1293\;(R^{2}=0.8091)$$ (17)

$$k=0.0079\varphi^{2}+0.2284\varphi +0.9051\;(R^{2}=0.9554)$$ (18)

$$k=-0.0329\varphi^{2}+0.4602\varphi -0.1158\;(R^{2}=0.6411)$$ (19)

As described above, Eq (3) becomes:

$$y=\frac{a}{1+e^{-k(\varphi )(t-t_{c})}}$$ (20)

The maximum *k* value of tall fescue, Kentucky bluegrass and perennial ryegrass is 1.1548, 1.6946 and 4.0940, respectively. This result indicates that the germination rate of perennial ryegrass is the quickest, and its cumulative seed germination rate first reaches the steady state. In this experiment, perennial ryegrass germinates too fast, while there are longer intervals between the observation points. Therefore, the test may change the value of *k* from high to low, and the *k* of perennial ryegrass is much higher than those of tall fescue and Kentucky bluegrass. The *k* of perennial ryegrass is higher than the others in the beginning of germination, which indicates that the response speed of perennial ryegrass is faster. In practice, perennial ryegrass can be used as a pioneer of mixed sowing, and the *k* of tall fescue is low, thus explaining why its reaction is less sensitive to water. This outcome could indicate that it possesses stronger drought resistance.

Based on Eq (20), a relationship can be obtained for the germination rate and irrigation water content; assuming that *y*_*optimal*_ = *βy*, *β* can be obtained, and the original data are modified to determine the optimal regression equations for the three turfgrasses (Tall fescue, Eq 21; Perennial ryegrass, Eq 22; and Kentucky bluegrass, Eq 23):

$$y_{optimal}=\frac{73.3556}{1+e^{-1.0617(t-1.5)}}\;(R^{2}=0.8314)$$ (21)

$$y_{optimal}=\frac{89.1278}{1+e^{-1.6614(t-1.5)}}\;(R^{2}=0.7142)$$ (22)

$$y_{optimal}=\frac{70.0540}{1+e^{-1.4610(t-1.5)}}\;(R^{2}=0.8659)$$ (23)

**Fig 4:**
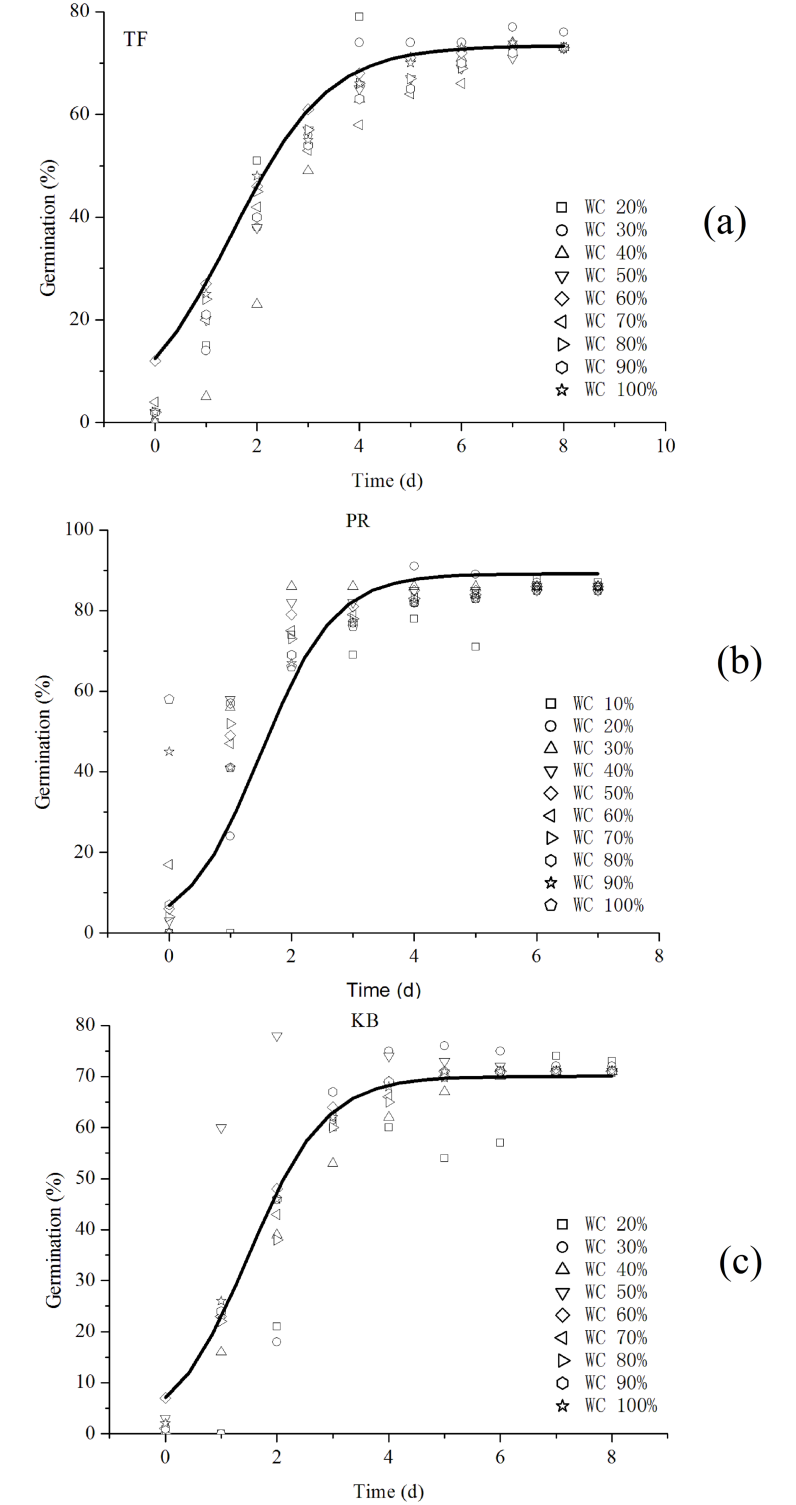
The optimal fitting curves of the cumulative germination in a range of Irrigation Water Contents (WC Indicates Water Content) for three turfgrasses. (A) Tall fescue (TF), (B) Perennial ryegrass (PR) and (C) Kentucky bluegrass (KB).

These three equations show the cumulative germination rate in response to the different sensitivities of water for the three turfgrasses. The optimal value of *k* is 1.0617 for tall fescue, 1.4610 for Kentucky bluegrass, and 1.6614 for perennial ryegrass. This result indicates that perennial ryegrass seeds are more sensitive to water than tall fescue and Kentucky bluegrass. Therefore, perennial ryegrass will be the fastest to reach the maximum value of the germination rate. Using the experimental *k* of the optimal water on its fitting curve that represents *k*, *t*_*c*_ is the time for optimal water from this experiment. In addition, the error of *a* may come from the fitting error of *k* and the estimation error of *t*_*c*_. Fig 4 shows the optimal fitting curves of the cumulative germination for the three turfgrasses, and it indicated that the slope increases with *k*. When *k* is growing, it would take less time to achieve the maximum value of the germination rate.

## Conclusions

Water content significantly affects the germination of turfgrass. Turfgrass has a high germination rate when the water conditions are appropriate. The results from the present experiment indicate that the seed germination function can describe the response of turfgrass seed germination to external water content variation and their sensitivities. This function can be used to quantitatively study the dynamic changes in seed germination under different water conditions. In addition, this function can confirm the characteristic parameters to express the sensitivities of seeds with water. The seed germination function has been tested with the data obtained. Both the function and the measured data showed that the variation of the cumulative seed germination rate is “slow—fast—slow”. At the beginning of germination, the cumulative germination rate increased quickly and gradually tended to remain steady. The cumulative germination rate increased rapidly as the water content increased. When the water reached a certain value, it reached its maximum value and tended to slightly and gradually decrease. The results indicated that there was an optimal water content that led to the seed germination rate reaching its maximum.

The germination of the turfgrass seeds was mainly affected by the rate of change of the water rather than the amounts of total water. Therefore, choosing appropriate irrigation methods is useful to maintain stable external water contents and high water use efficiency to meet the needs of seed germination. Based on this study, the optimal value of *k* was 1.6614 for perennial ryegrass, which implies that perennial ryegrass seeds are more sensitive to water content than those of tall fescue and Kentucky bluegrass. The *k* value of tall fescue was lowest, which suggested that tall fescue is less sensitive to water. However, tall fescue exhibits more drought resistance than the other two types of turfgrasses. Based on the seed germination function, further research should be conducted using experiments under different cultivation media conditions to improve turfgrass seed germination models through field experiments.
